# The coracobrachialis muscle: typical morphology, accessory forms, and the issues with terminology

**DOI:** 10.1007/s00276-023-03258-w

**Published:** 2023-11-07

**Authors:** George Triantafyllou, Konstantinos Natsis, Georgi P. Georgiev, Christos Koutserimpas, Łukasz Olewnik, George Tsakotos, Nicol Zielinska, Maria Piagkou

**Affiliations:** 1 Department of Anatomy, School of Medicine, Faculty of Health Sciences, National and Kapodistrian University of Athens, 75 Mikras Asias Str., Goudi, 11527 Athens, Greece; 2 Department of Anatomy and Surgical Anatomy, School of Medicine, Faculty of Health Sciences, Aristotle University of Thessaloniki, Thessaloniki, Greece; 3 Department of Orthopedics and Traumatology, University Hospital Queen Giovanna-ISUL, Medical University of Sofia, Sofia, Bulgaria; 4 Department of Orthopaedics and Traumatology, “251” Hellenic Air Force General Hospital of Athens, Athens, Greece; 5 Department of Anatomical Dissection and Donation, Chair of Anatomy and Histology, Medical University of Lodz, Lodz, Poland

**Keywords:** Coracobrachialis muscle, Variation, Morphology, Accessory muscle, Head, Variant, Anatomy

## Abstract

The coracobrachialis muscle (CB) represents one of the anterior arm compartment muscles. It has been defined by classic anatomy textbooks and old papers, as a muscle of one belly arising from the coracoid process tip and partially from the tendon of the biceps brachii short head, and inserted into the humeral shaft, above the bone’s midpoint. However, recent cadaveric studies have confirmed that in the majority of cases, the CB is a two-headed muscle consisting of a superficial and a deep head. This finding has caused confusion regarding the terminology of CB’s morphology. Typical CB morphology, according to recent data should not be considered the muscle of one belly, but the two-headed muscle. In particular, the musculocutaneous nerve’s (MCN) course plays an important role in defining the CB morphological characteristics. If the MCN courses medially to the CB, with no signs of penetration after dissection, it can be concluded, that the CB is composed of one head; otherwise, if the muscle is composed of two or more heads, the MCN will courses between the CB bellies. In conclusion, it is of paramount importance to adopt common-universal terminology. Hence, considering recent findings, if the CB origin and/or the insertion differs from the typical anatomy, an “atypical CB” is the proper definition of the muscle, while if this “atypical CB” coexists with a typical CB, the terminology “accessory CB” may be used.

The coracobrachialis muscle (CB) is one of the muscles of the anterior arm compartment. Classic anatomy textbooks refer to it, as a muscle of onebelly arising from the coracoid process (CP) tip and partially from the tendon of the biceps brachii short head, and inserted into the humeral shaft, above the bone’s midpoint [18]. Nevertheless, the CB usually is separated into superficial and deep parts and rarely has an additional part, the so-called accessory part (head or muscle), as reported by many authors [8, 12, 18, 20].

Wood [21] was one of the first to report that the CB was separated into upper and lower fibers, while the musculocutaneous nerve (MCN) passed between the two portions. He first defined CB morphological variants, such as the CB longus and brevis (CBL and CBB) [21]. Based on his observations [21], Testut [16] in his study very rarely identified the CB division into two portions [16]. Similarly, Le Double [9] identified a division of CB into two portions in a unique specimen, while Macalister in his manuscript did not identify this “malformation” of the muscle [10]. Testut and Latarjet in their book [17] reported cases, in which the MCN coursed between the CB two portions. Mori et al. [10] systematically studied the CB complete or partial separation into superficial and deep layers and justified this division, by the muscle’s developmental background. Tountas and Bergman [18] quoted that the CB is believed to be formed of three distinct parts, arising from the CP, and inserted proximally, medially, and distally into the medial surface of the humerus. The proximal part is the most deeply situated, and the distal one is the largest and most superficial. In humans, the middle part is the most constant and is usually accompanied by a portion of the distal part, with the MCN coursing between the two parts [18]. These two parts constitute the CB most commonly identified CB morphology. The CB variants are aberrations (usually extensions) of its proximal and/or distal part, as well as the CB joining (fusion) to the adjacent musculature [18]. CB innervation usually derives from the MCN, which also penetrates the muscle’s belly [18].

Based on the above-mentioned data, is the CB of one belly, the typical muscle’s morphology?

## The findings from cadaveric morphological studies

### CB morphology

El-Naggar [2] investigating the CB morphology in 36 cadavers, discovered a two-headed CB consisting of a superficial and a deep head, in most of the cases. Interestingly, El-Naggar reported that the presence of a two-headed CB could be attributed to the variable degrees of fusion of its ancestral two heads, due to the fact that in some mammals, the CB is composed of two muscular heads [2]. This finding was later confirmed by Ilayperuma et al. [5], who identified a two-headed CB in 83.33% (260 upper limbs). Similarly, Piagkou et al. [13] recorded a two-headed CB (typical morphology) in 62.96%, after dissecting 27 cadaveric arms. These three cadaveric studies [2, 5, 13], concluded that the CB typical morphology consists of two heads, although Szewczyk et al. [15] found a single-headed CB, as the most common form (49.5%), in accordance with the classic and old anatomy textbooks. In the Szewczyk et al. [15] study, the two-headed CB occurred in 42.6% of cases.

### MCN relationship to the CB morphology

Vallois [19] first pointed out cases of CB in which the MCN didn’t penetrate its parenchyma. Nevertheless, Testut and Latarjet reported that they did not identify the MCN medial course in relation to the CB [17]. Recently, Ilayperuma et al. [5], Piagkou et al. [13], and Szewczyk et al. [15] observed that if the muscle is composed of two or more heads, the MCN coursed between the CB bellies. If the MCN courses medially to the CB, with no signs of penetration after dissection, it can be concluded, that the CB is composed of one head. This discovery allows us to make a safe prediction about the CB morphology, after identifying the MCN origin and course. The MCN pathway through CB layers (referred to as “penetration”) verifies the CB division into two or more segments. This finding supports Koizumi’s [7] assertion that the CB is a composite muscle that receives input from at least two distinct nerves. Any other muscle morphology may result in alterations in the MCN route and neural supply of the muscle. According to Georgiev [3], to accept correctly variant muscle, three important issues are essential: (1) the muscle’s location; (2) the muscle’s insertions; and (3) the muscle’s function. In our opinion, this statement could be extended, and a fourth point can be added, the close relationship of the muscle with the surrounding nerve.

### Issues of terminology concerning the CB morphology

Vrzgula et al. [20] described an “accessory CB” located anterior to the “typical CB”, and the MCN penetrated the “typical CB”. They asserted that the identified CB variant was either a muscle duplication or a complete separation of it [20], according to Mori et al. [10] theory. Kumar et al. [8] described an “accessory CB” that originated from the CP, joined the biceps brachii short head, and was inserted into the medial epicondyle of the humerus [11]. The MCN coursed between typical and accessory CB [8]. Similarly, Paraskevas et al. [12] identified an “accessory CB” composed of two heads, a superficial and a deep one, both originating from the CP tip. The superficial head had an atypical insertion into the brachial fascia and the medial intermuscular septum, but no further information regarding the MCN course was provided.

To build a consensus and uniform terminology concerning the CB morphological variants, and related neural supply, it is important to first identify the typical CB (superficial and deep) heads, the MCN relationship with the CB, and after recording the accessory (supernumerary or variant) heads of the muscle, as well as the MCN variant course. It is crucial to identify these variants in clear and informative dissection and depict them in high-quality images as Georgiev [4] aptly noted in his letter. Thus, cadaveric studies gain more and more relevance regarding the identification of such variants. In scoping to properly define a CB variant, the following aspects of the muscle must be considered: The origin and muscle’s insertion, the MCN course between the muscle heads or the medial course in relation to the muscle, and the presence of supernumerary or accessory head(s). If the CB origin and/or the insertion differs from the typical anatomy, an “atypical CB” is the proper definition of the muscle (atypical or aberrant means diverging from the usual type) [6]. If this “atypical CB” coexists with a typical CB, the terminology “accessory CB” can be used (accessory means additional muscle, aiding or contributing in a secondary way) [6] because the identified muscle is a slightly different muscle that coexists with the typical one (coracobrachialis accessorius [18]). Sookur et al. [14] and Desimpel et al. [1] pointed out that the accessory muscles are variants representing additional distinct muscles that are encountered along with the normal complement of muscles. Muscle variants may consist of the absence of a muscle, supernumerary muscles, deviation from the normal course, or an anomalous origin or insertion [1]. An example of an atypical or variant CB is the CBL, with a distal insertion, closer to the elbow joint [21, 22]. The identification of supernumerary or accessory or variant head(s) should be done concerning the CB typical morphology and MCN course. If a variant head(s) of the CB has/have a typical (or close to the typical) origin and insertion and coexists with the typical CB, the head(s) should be called “accessory or supernumerary CB head(s)” rather than “accessory CB” because the typical morphology of CB exists, thus it is not an accessory muscle but a variant (accessory) component of the CB.

In conclusion, taking into consideration the recent findings from cadaveric studies, the CB typical anatomy should be considered the two-headed muscle, consisting of a superficial and a deep head that also seems to be the prevalent morphology. The MCN course plays an important role in defining the CB muscle’s morphology and should be studied if possible. Moreover, careful use of the term “accessory” muscle should be made, since the typical morphology should always be present for this term to be used.

## Data Availability

Data and material related to the report will be available with the corresponding author for further reference.
